# Contemporary diets of walruses in Bristol Bay, Alaska suggest temporal variability in benthic community structure

**DOI:** 10.7717/peerj.8735

**Published:** 2020-03-19

**Authors:** John M. Maniscalco, Alan M. Springer, Katrina L. Counihan, Tuula Hollmen, Helen M. Aderman, Moses Toyukak, Sr.

**Affiliations:** 1 Science, Alaska SeaLife Center, Seward, AK, United States of America; 2 College of Fisheries and Ocean Sciences, University of Alaska—Fairbanks, Fairbanks, AK, United States of America; 3 Qayassiq Walrus Commission, Dillingham, AK, United States of America

**Keywords:** Pacific walrus, *Odobenus rosmarus divergens*, Diet, qPCR, Bristol Bay, Alaska

## Abstract

**Background:**

Pacific walruses (*Odobenus rosmarus divergens*) are a conspicuous and important component of the Bristol Bay ecosystem and human social systems, but very little is known about walrus ecology in this region, principally their feeding ecology. The present work provides contemporary data on the diets of walruses at four haulout locations throughout Bristol Bay between 2014 and 2018.

**Methods:**

We analyzed scat and gastrointestinal tract samples from these animals using quantitative polymerase chain reaction to amplify prey DNA, which allowed for diet estimates based on frequencies of prey item occurrence and on the relative importance of dietary items as determined from DNA threshold cycle scores.

**Results:**

Diets were highly diverse at all locations, but with some variation in composition that may be related to the time of year that samples were collected (summer vs. autumn), or to spatial variability in the distribution of prey. Overall, polychaetes and tunicates had the highest frequencies of occurrence and relative abundances in 2014–15, but a major change in diet appears to have occurred by 2017–18. While some sample sizes were small, diets in these later years contrasted sharply, with a greater prevalence of sea cucumbers and mollusks, and reduced importance of decapods and fishes compared to the earlier years. Prey identified in scat samples from one collection site also contrasted sharply with those reported from the same location in 1981. The apparent temporal shifts in walrus prey may represent a changing benthic ecosystem due to warming waters in recent decades.

## Introduction

Bristol Bay is a biologically rich expanse of marine habitat over the continental shelf in the southeastern Bering Sea. Like most high-latitude ecosystems, it exhibits strong seasonal pulses of productivity that support complex food webs. Several upper trophic level predators including the Pacific walrus (*Odobenus rosmarus divergens*) inhabit Bristol Bay throughout the year. The walruses here are all males and are especially abundant during the non-breeding season between May and October, feeding across the bay and hauling out by the thousands to rest in predictable locations (Fay & Lowry, 1981; Jay & Hills, 2005).

The walruses inhabiting Bristol Bay are an important traditional subsistence and cultural resource of area residents (Fall & Chythlook, 1998), but concerns over changes in their abundance and risk to disturbance at local haulouts (Wilson & Evans, 2009), combined with several potential anthropogenic threats, have prompted the need for studies of their ecology in this region. Potential threats to walruses in Bristol Bay include human-induced disturbances at their haulouts and foraging areas, disruptions of prey resources by commercial trawl fishing operations, and contaminants released from prospective oil, gas, and mineral exploration and extraction activities proposed in the region (MacCracken, 2012). Yet, climate change and its effects on marine environments are potentially the greatest long term threat to the Bering Sea ecosystem. Warming waters and diminishing sea ice both have direct and indirect effects on the structure of marine communities, including the abundance, productivity, and distribution of many marine organisms, including those that are important prey for walruses (Grebmeier et al., 2006; Mueter & Litzow, 2008; Sheffield & Grebmeier, 2009; MacCracken, 2012).

Pacific walruses are typically benthic feeders, preying primarily upon bivalves, gastropods, crabs, and various worms (Fay, 1982; Sheffield & Grebmeier, 2009). However, data regarding walrus diets in Bristol Bay are limited. Information comes from Traditional Ecological Knowledge of residents who hunt walruses and from four animals collected in southern Bristol Bay in February–April 1981 (Fay & Lowry, 1981). Additional collections of stomach contents from 73 walruses on the continental shelf of the southeastern Bering Sea occurred to the west of Bristol Bay on three occasions in winter-spring between 1962 and 1976, and from 180 animals in February–April 1981 greater than 70 km west of Cape Newenham (Fay & Lowry, 1981). Elsewhere, diets of walruses are known to differ substantially across spatial scales comparable to these (Sheffield & Grebmeier, 2009). Thus, previous understanding about the prey resources that support walruses in Bristol Bay at any time during the year has been sparse, and no information has been available on diets during summer and autumn. The research presented here was undertaken to establish contemporary information on the diet of walruses in Bristol Bay so that impacts that may occur due to environmental change forced by trawl fisheries, future oil and gas exploration and development, and climate change can be identified and mitigated to conserve walruses in their own right, and their value as an important resource to area residents.

We used quantitative polymerase chain reaction (qPCR) analysis of prey DNA in feces (scat samples; Bowles & Trites, 2013) and gastrointestinal tracts (e.g., Tollit et al., 2009) to characterize the diets of walruses. Samples from feces and gastrointestinal tracts provide a representation of diet within the previous hours to less than a day due to rapid food passage times of walruses (Fisher, 1989; Kastelein et al., 2003). The use of qPCR provides estimates of the relative contribution of different prey items in the diet and is superior to examination of stomach contents (Deagle et al., 2005; Bowles & Trites, 2013) because the latter method is highly subject to biases associated with differential digestion times of prey species (Sheffield et al., 2001). In addition, walruses typically consume only soft tissues of their prey, leaving hard parts behind (Sheffield & Grebmeier, 2009). By utilizing modern DNA-based diet identification, we were able to determine important components and recent changes in the diets of walruses inhabiting Bristol Bay.

## Materials & Methods

### Sample collections

**Figure 1:**
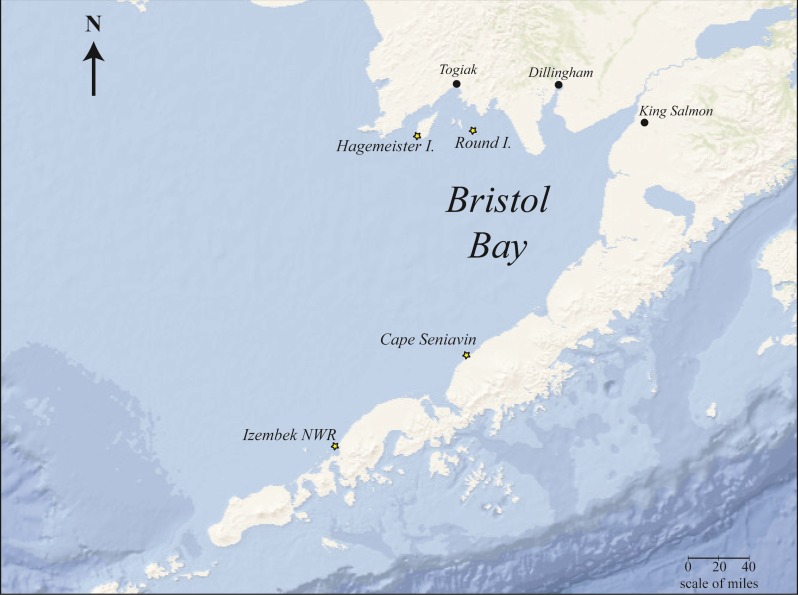
Bristol Bay, Alaska study area showing Pacific walrus sample collection sites (starred).

Samples were collected for diet analysis from four locations around Bristol Bay (Fig. 1) during the years 2014–2015 and one location (Round Island) in 2017–2018. Not all locations were sampled in every year. These samples included 52 scat samples and four gastrointestinal tract samples (Table 1).

**Table 1:** Samples collected for qPCR analysis of diet for Pacific walruses in Bristol Bay, Alaska.

Month/year	Location	Sample type	No. samples
Oct 2014	Hagemeister Is.	Scat	8
Oct 2014	Hagemeister Is.	^a^G.I. tract	3
Jun 2015	Round Is.	Scat	4
Jul–Aug 2015	Izembek	Scat	7
Sep 2015	Cape Seniavin	Scat	17
Oct 2015	Hagemeister Is.	^a^G.I. tract	1
Jun–Jul 2017	Round Is.	Scat	9
May–Jul 2018	Round Is.	Scat	7

**Notes.**

a Gastrointestinal tract from animals harvested in native Alaskan hunt.

Estimates of predator diets using qPCR require a library of known or suspected prey with identified DNA sequences. Collaborating with the National Marine Fisheries Service, we obtained walrus prey samples from their annual bottom trawl fishery surveys in Bristol Bay and the eastern Bering Sea during 2014 and 2015. Fishes and other organisms that were obtained in these surveys are known to occur in diets of walruses in the eastern Bering Sea and elsewhere (Fay & Lowry, 1981; Sheffield & Grebmeier, 2009; Wilson & Evans, 2009). A total of 203 individual specimens from 67 prey species were obtained and analyzed for DNA sequencing.

Individual walrus scat samples were collected opportunistically from the haulouts after walruses entered the water or moved away from the collection sites. Samples we collected were discrete blobs of the same color and consistency to help avoid mixed samples from multiple individuals. Stomach, and upper and lower intestinal track contents for DNA analysis were obtained from walruses hunted at Hagemeister Island in 2014 and 2015. All samples were stored in 100% ethanol and frozen at 20 °F prior to analysis.

### Laboratory analysis

DNA was extracted from the 67 potential prey species for use as controls in qPCR and for primer design. Universal primers (Bowles & Trites, 2013; Deagle et al., 2013) were used to amplify and sequence a portion of the 16S rDNA using PCR in species that did not have 16S sequences available in GenBank. 16S rDNA sequences were obtained for 20 species. Previously published amphipod (Deagle et al., 2007) and echinoderm primers (Jarman, Redd & Gales, 2006) were available, and five additional family-specific primer sets for seals, bivalves, gastropods, decapods, and fish were designed for qPCR for broad prey identification. Forty-eight species-specific primer sets were designed for qPCR that included: 13 bivalve primer sets, five gastropod primer sets, six crab primer sets, two shrimp primer sets, 12 fish primer sets, and 10 primer sets for other species such as anemones, burrowing worms, tunicates, and sea cucumbers (Appendix S1). Primers were designed based either on published sequences or sequencing of trawl samples. The specificity of designed primers was tested *in silico* with sequences from GenBank. The primer sets were tested with genomic DNA from related species to ensure no cross-reactivity occurred. Amplified products from each primer set were sequenced and analyzed with BLAST to confirm specificity. Some species were too closely related to develop a species-specific primer set. Primers that detected more than one species are shown in Appendix S1.

Scat and gastrointestinal samples collected for prey identification were rinsed through 0.5 mm mesh sieves to remove any hard pieces and into 50 ml tubes containing 15 ml of 100% ethanol. DNA was extracted using a Qiagen DNA Stool Kit. Extracted DNA was initially run with 13 different primer sets and all samples were run separately. Cycling conditions included initial denaturization at 95 °C for 20 s, followed by 40 cycles of 95 °C for 15 s, annealing for 15 s, and extension at 72 °C for 1 min. Annealing temperature varied based on the primer set. Eleven primer sets were used to identify amphipods, sea cucumbers, sea squirts, spoon worms, polychaetes, burrowing worms, sea anemones*,* and harbor and spotted seal DNA present in the samples. The other three primer sets were designed to detect 16S rDNA from broad groups of mollusca, decapoda, or fish. Any samples that were positive for mollusca primers were run with an additional 17 primer sets to detect specific bivalve and gastropod species and genera. Samples that were positive for decapoda primers were run with an additional eight primer sets to identify specific crabs and shrimps. Samples that were positive for the fish primers were run with 12 primer sets to detect specific groundfish and forage fish genera. The majority of these primers were designed to detect mitochondrial 16S rDNA, however some primers targeted the small ribosomal subunit, 18S, because that was the only sequence data available in GenBank. The full list of primers used in this study is provided in Appendix S1. Total quantities of DNA targeted and not targeted in each sample run were used as the denominator for calculations of concentrations of each prey type (see *Data Analysis*).

Captive walrus scat samples (*N* = 25) were received from three zoos and aquaria and analyzed to validate the use of qPCR for detecting and quantifying specific prey types in their feces. Captive walruses were fed a diet consisting of recorded proportions of herring, capelin and clams. DNA was amplified using broad bivalve and fish primers and specific clam, capelin and herring primers. The broad fish primers were designed specifically for Pleuronectiformes, which is the primary order of fish that walrus in Alaska are thought to feed on. Herring and capelin belong to different orders (Clupeiformes and Osmeriformes, respectively), so the broad primers will tend to amplify DNA from those species only when it was present in higher concentrations. It was not a primary goal of this study to conduct a strictly controlled experiment to estimate correction factors for amounts of prey fed to walruses based on relative proportions of DNA detected in their scat samples. Rather, the main intent of this captive walrus work was to assess prey detection and lack of detection for items fed and not fed to these animals. Nevertheless, 17 scat samples were associated with individual captive walruses whose dietary food proportions consisting of herring, capelin and/or clams were known within the previous days. These data were used as an ad hoc test for correlations between proportional DNA content in scat samples and the proportions of foods fed to these walruses.

### Data analysis

We categorized and analyzed the diets of walruses in Bristol Bay spatially based on collection location, and temporally based on collection years (2014–15 vs. 2017–18) because initial explorations of the data suggested a change in diet between these two periods. Results are generally presented using descriptive and exploratory statistics based on frequency of occurrence (FO), and threshold cycle (C_t_) values calculated by the qPCR analysis and explained below. We used percent FO to assess the relative presence of various prey items in the diet, which was calculated based on the additive presence of each particular prey type in each sample divided by the total number of samples collected per location and time period then multiplied by 100. The Shannon index of diversity (Hutcheson, 1970; Zar, 1999) was calculated for each study location as ${H}^{{}^{{^{\prime}}}}=-{\mathop{\sum }\nolimits }_{i}^{k}{p}_{i}\ln {p}_{i}$, where *p* is the fraction of each prey type *i* observed in the samples collected at each location, *k* is the number of prey types, and ln is the natural logarithm.

Spatial and temporal variation in the FO data were explored using a principal components analysis. We also used C_t_ values to calculate an index of the relative importance of various prey items found in walrus samples. C_t_ is defined as the number of cycles necessary for the fluorescent signal to exceed threshold background levels. Lower C_t_ values indicate larger quantities of the target DNA. Therefore, to estimate the proportion of different prey groups and species in the diet of walruses, inverse functions of the targeted, DNA-specific C_t_ values per ng total DNA (targeted and non-targeted) in each sample were converted into percentages relative to each other. For the captive walrus data, a Wilcoxon signed rank paired-sample test was used to determine if the proportion of prey DNA in scat samples was correlated with the proportion of foods fed to these animals. For samples from Bristol Bay walruses, Bray–Curtis distances (dissimilarity indices) were computed with C_t_ data to test for differences between sites with a permutational multivariate analysis of variance (PERMANOVA; Anderson, 2001), which is analogous to a traditional multivariate ANOVA but does not require multivariate normality. A test of homogeneity of dispersion was also necessary to determine if significant results were due to site differences, unequal dispersion among the sites, or a combination of these. We had no reason to suspect that DNA in gastrointestinal tract samples from Hagemeister Island would be different from that in scat samples from this location because of the short (<1 day) food passage times of walruses (King et al., 2008). Nevertheless, we performed a PERMANOVA between these sample types to determine if significant differences in diet results existed. Significant results were explored further with a pairwise PERMANOVA controlled for false positive discovery rate (Benjamini & Hochberg, 1995) and graphically with multidimensional scaling (MDS) of Bray-Curtis distances, which assigned each sample a location in two-dimensional space relative to all other samples.

Statistical analyses and comparisons were conducted in R (R Core Team, 2017) with means presented ± standard error (SE) when comparing means among sites or standard deviation (SD) for the means of all individual samples. We conducted a centered and scaled principal components analysis using the prcomp function in R. Shannon indices of diversity, PERMANOVA calculations, and MDS were conducted using package vegan (Oksanen et al., 2019).

## Results

### Captive validation

**Figure 2:**
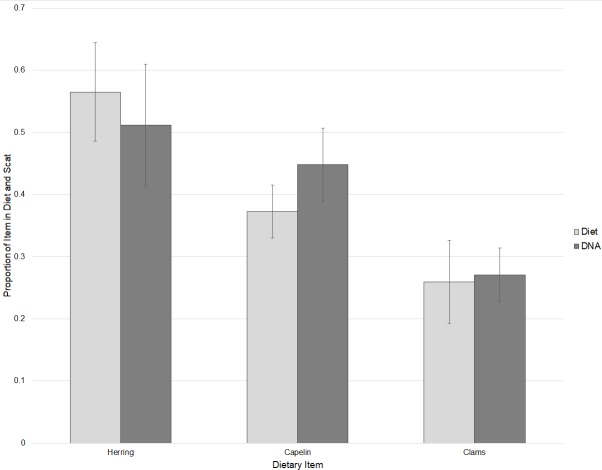
Comparisons of relative proportions of dietary items fed to captive walruses and the mean proportions of these items detected in their scat samples based on threshold cycle (*C*_*t*_) values ± SE.

DNA of clams, herring, and capelin was detected in the diets of captive walruses fed these foods. As expected, the broad fish primer that was designed primarily for flatfishes detected very little fish DNA in the captive walruses. However, primers specific to herring (*Clupea pallasii*) and capelin (*Mallotus villosus*) did substantially amplify DNA of these items in their diets. Based on C_t_ values, relative proportions of herring, capelin, and clam DNA detected in scat samples were not statistically different to proportional amounts of these items fed to the walruses (*P* = 0.740) with some individual variation (Fig. 2). Captive walrus scat samples were also tested for DNA of seals (*Phoca spp*.), in addition to broad primers for gastropods and decapods, and specific primers for sea cucumbers (*Cucumaria spp*.), a marine annelid (*Lumbrineris sp*.), and a tunicate (*Styela rustica*). These are all prey items that occur in the diets of wild Bristol Bay walruses. With the exception of one individual walrus sample which tested positive for decapods and sea cucumber, none of these items were identified in the captive walrus scat samples.

### Diets of wild walruses

**Figure 3:**
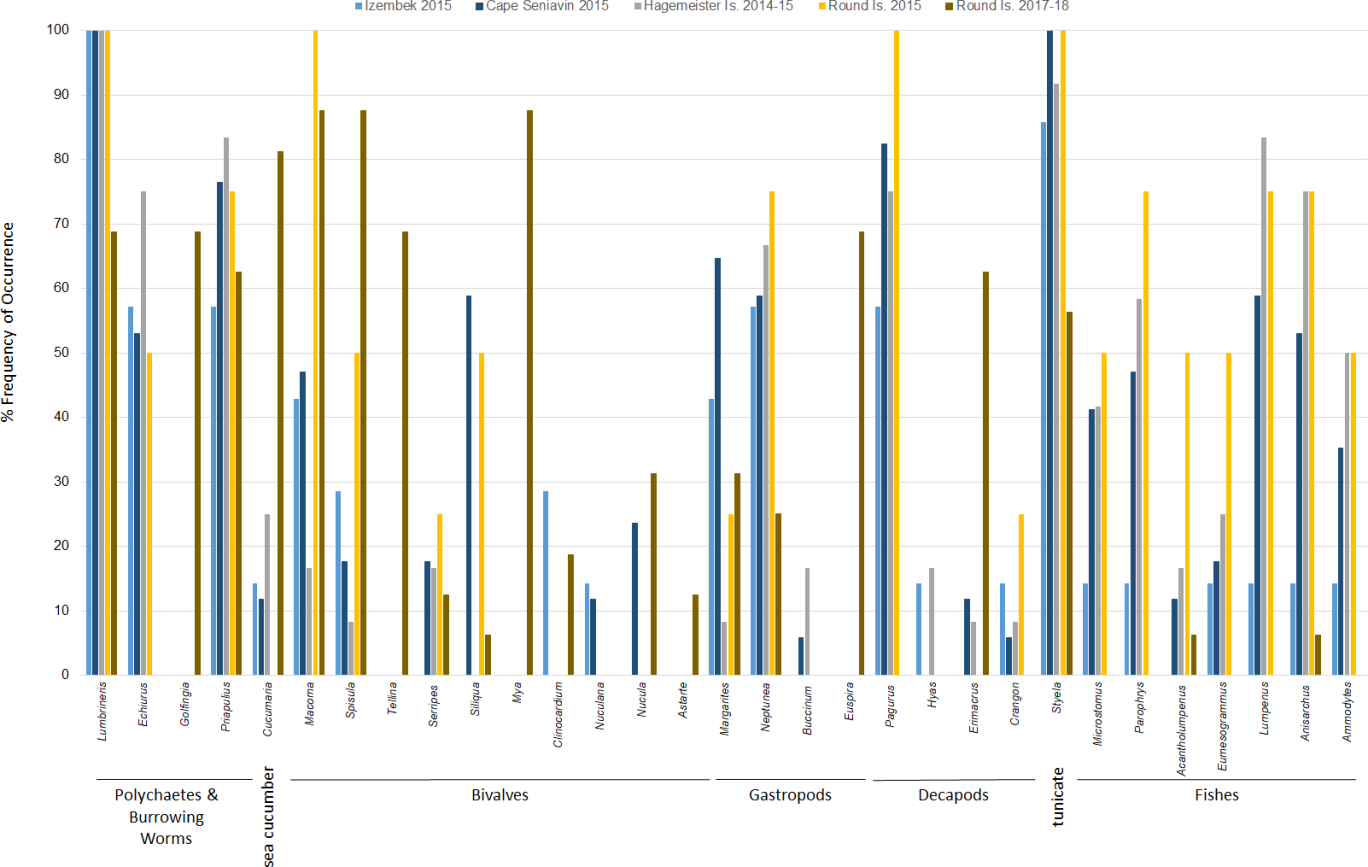
Frequency of occurrence of Pacific walrus prey genera detected in >5% of samples (31 of 36) collected at four different sites within the study area and pooled by study site.

Diets of walruses in Bristol Bay were represented by a minimum of 36 genera or species ($\bar {x}=9.10$ ± 3.61 SD per sample; $\bar {x}=22.4$ ± 1.03 SE per site) detected in scat and GI tract samples over the years 2014–15 and 2017–18 combined (Fig. 3). We did not find a significant difference between prey DNA in gastrointestinal tracts and DNA in scat samples from walruses at Hagemeister Island (*P* = 0.318). Therefore, scat and gastrointestinal samples were lumped together for this location. Shannon indices of diversity ranged narrowly at four of the five sites, from *H*′ = 2.81 at Round Island in 2017–18 to *H*′ = 2.88 at Hagemeister Island in 2014–15, but diets were most diverse at Cape Seniavin in 2015 (*H*′ = 3.02).

**Figure 4:**
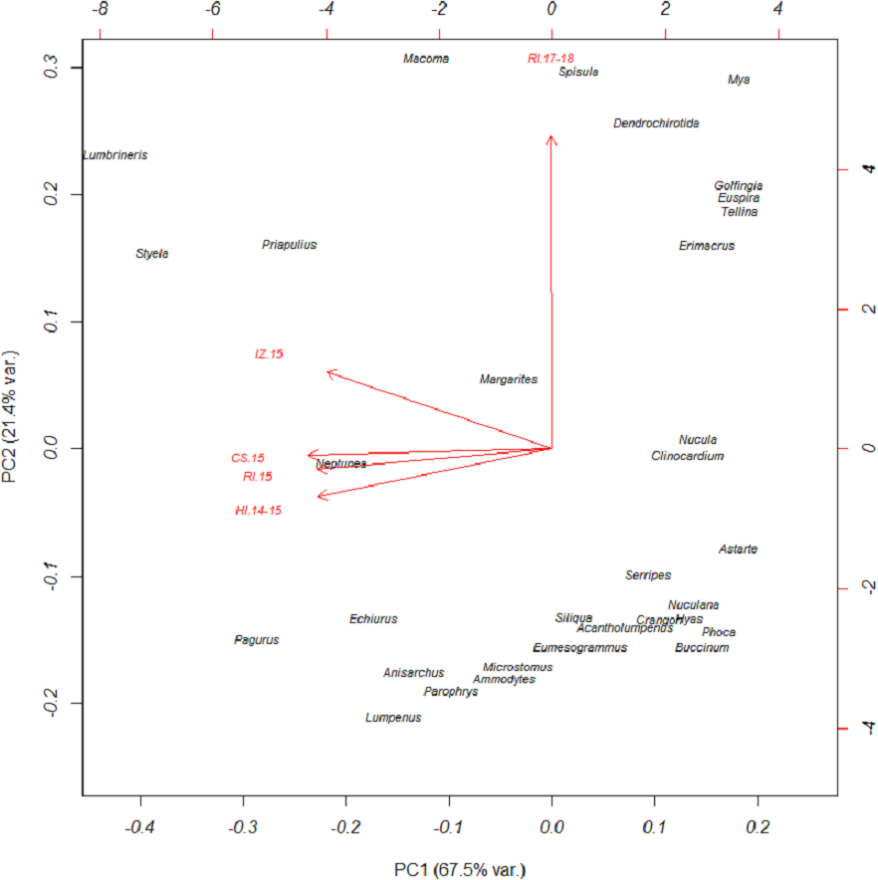
Principal components plot of walrus diet items. Principal components plot based on percent frequency of occurrence of dietary items from Pacific walruses in Bristol Bay, Alaska. Arrow vectors represent the loadings by sample area (HI.14-15 = Hagemeister Island, RI.15 = Round Island in 2015, CS.15 = Cape Seniavin, IZ.15 = Izembek NWR, RI.17-18 = Round Island in 2017–2018).

Based on FO at all sites and years, benthic worms (primarily *Lumbrineris* and *Priapulus spp.*), and tunicates (*Styela rustica*) were among the most common prey in the diets of Bristol Bay walruses. Sea cucumbers were more common and fishes less common in the diets of walruses at Round Island in 2017–18 compared to Round Island in 2015 or any other location from 2014–15. Bivalves also generally appeared more often in samples from Round Island in 2017–18 compared to samples from there and other locations in 2014–15. Hermit crabs (*Pagurus spp*.) were the most common decapod consumed, but these were not seen in the diet of walruses at Round Island in 2017–18. Rather, the moon snail (*Euspira pallida*), horsehair crab (*Erimacrus isenbeckii*) and a variety of bivalves were widely detected in their diet in these later years (Fig. 3). All of these differences in diet were reflected in the principal components analysis, where Round Island 2017–18 was much different than any other grouping based on the first two principal components that explained 89% of the variation in the data (Fig. 4).

Species of commercial and food web importance that were tested for DNA sequences but not detected include red king crab (*Paralithodes camtschaticus*), snow crab (*Chionoecetes opilio*), northern prawn (*Pandalus borealis*), flathead sole (*Hippoglossoides elassodon*), northern rock sole (*Lepidopsetta polyxystra*), halibut (*Hippoglossus stenolepsis*), and capelin. There was a small signal of yellowfin sole (*Limanda aspera*) in two samples from Hagemeister Island, and limited detection of seals (two samples from Cape Seniavin in 2015) and Pacific herring (one sample from Round Island in 2018). Also not detected were amphipods (*Gammarus spp*.), catworms (*Nephtys spp*.), common sea anemone (*Metridium senile*), and melon-shape whelk (*Pyrulofusus melonis*), all of which are known or suspected prey types of walruses elsewhere (Fay & Lowry, 1981; Sheffield & Grebmeier, 2009).

**Figure 5:**
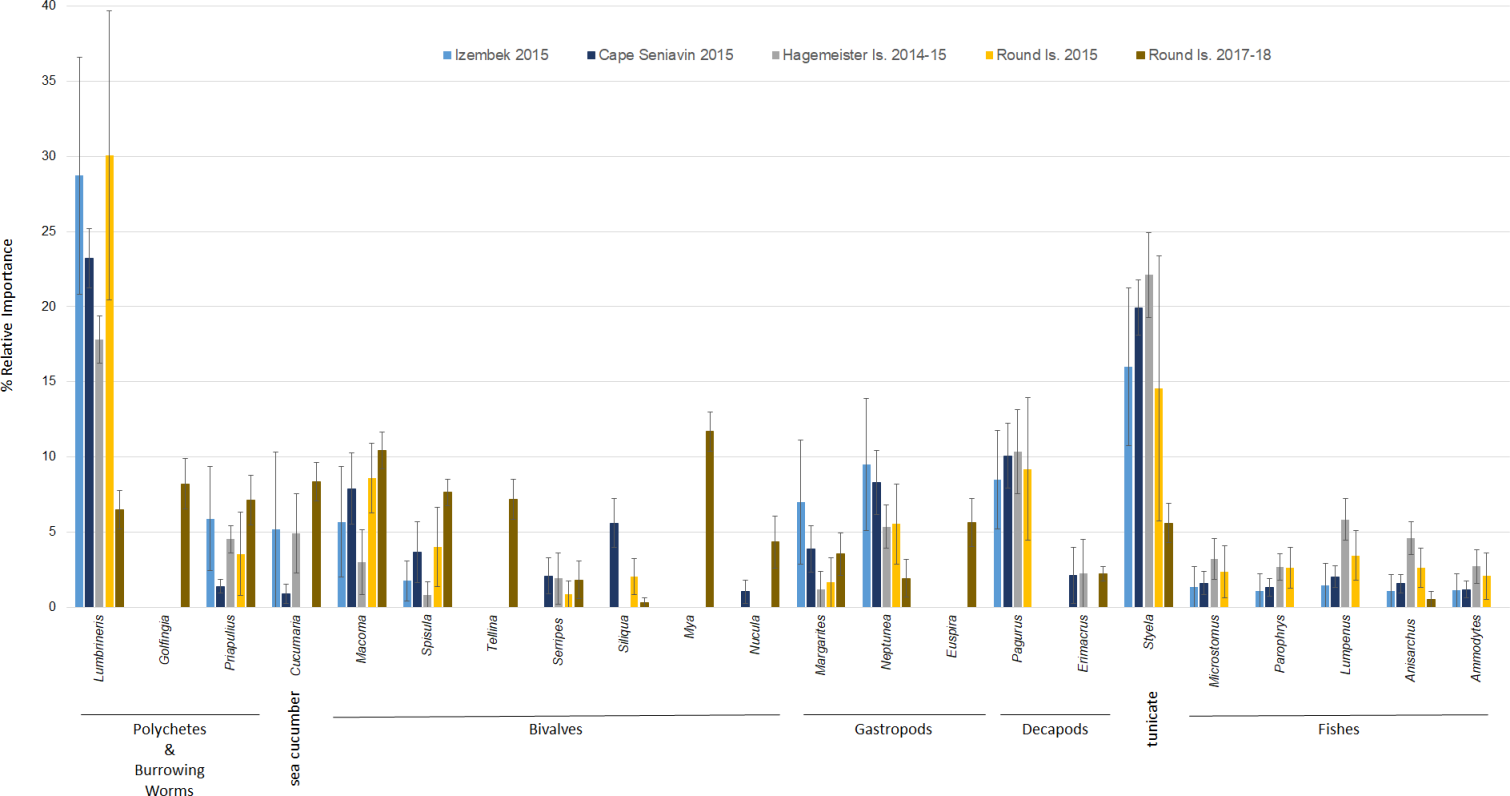
Relative abundance (>1%) of prey items in the diets of Pacific walruses based on *C*_*t*_ values ± SE pooled among study sites.

In terms of relative abundance based on C_t_ values, *Lumbrineris* polychaetes and tunicates were the most important species at all locations except Round Island in 2017–18, where there was a stronger influence of bivalves, sipunculid burrowing worms (*Golfingia sp.*), and sea cucumbers (Fig. 5). Margarite sea snails (*Margarites spp*.) and whelks (*Neptunea spp*.) were important gastropods at most locations, except the moon snail (*Euspira pallida*) was favored at Round Island in 2017–18. As with the FO data, decapods, especially hermit crabs (*Pagurus spp*.), were a meaningful component of walrus diet at all locations in 2014–15, but were absent at Round Island in 2017–18. Fishes were also somewhat important in the diets at Hagemeister Island and other locations, but negligible at Round Island in 2017–18.

**Figure 6:**
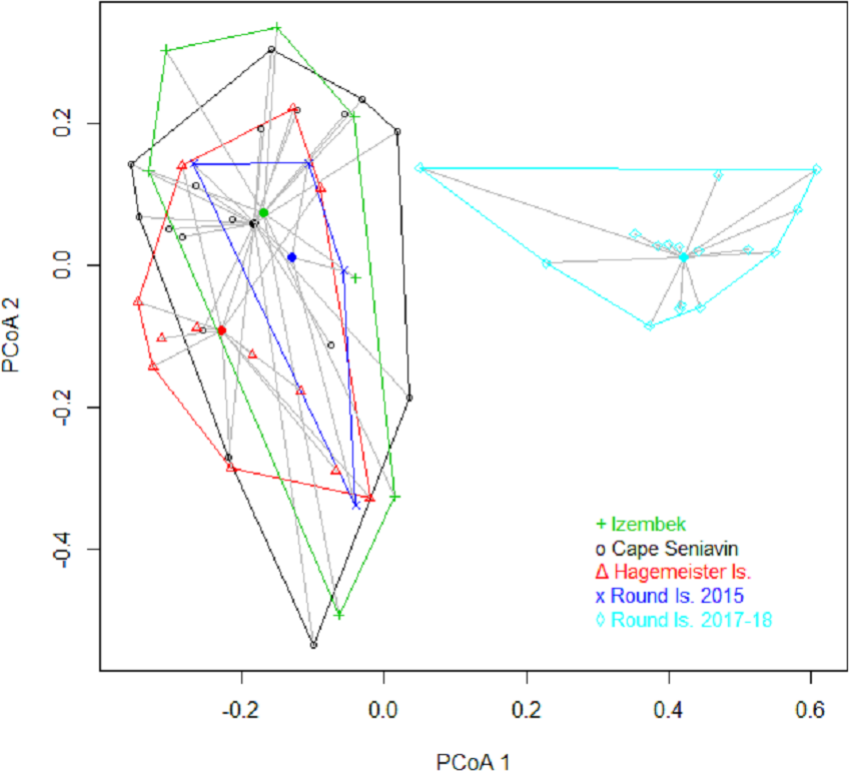
Plot of the first two principal coordinates in a multidimensional scaling analysis based on Bray–Curtis dissimilarity matrix of Pacific walrus diets in Bristol Bay, Alaska.

Results from the PERMANOVA on Bray-Curtis distance values of relative C_t_ scores were highly significant (*P* = 0.001) with no indication of unequal dispersion (*P* = 0.451). Paired comparisons showed that Round Island in 2017–18 was significantly different from all other sites (Table 2), and indeed exhibited no overlap with other sites based on distance metrics (Fig. 6). The only other significant difference that appeared in the paired comparisons was between Hagemeister Island and Cape Seniavin (Table 2).

**Table 2:** *P*-values of pairwise comparisons of Pacific walrus diets using PERMANOVA on a Bray–Curtis distance matrix.

	Cape Seniavin	Hagemeister Is.	Izembek	Round Is. 2015
Hagemeister Is.	0.016	–	–	–
Izembek	0.254	0.203	–	–
Round Is. 2015	0.586	0.152	0.902	–
Round Is. 2017–18	0.003	0.003	0.003	0.005

## Discussion

This study was undertaken to obtain contemporary data on the diets of walruses in Bristol Bay using modern DNA-based diet analysis. qPCR analysis using taxon- and species-specific primers to estimate diet composition has been shown to provide results similar to other modern DNA-based analyses such as high-throughput sequencing (Murray et al., 2011), and PCR has previously been shown to be an effective tool for identifying prey remains in scat samples of wild walruses (Bowles & Trites, 2013).

The qPCR analysis of captive walrus diet provided an opportunity to examine proximate variations between diet and scat. While we were unable to conduct a strictly controlled experiment to estimate correction factors for prey items fed to captive walruses, we did find that dietary proportions fed to those animals were generally equivalent to proportions of DNA in their scats. Variation in the findings of the captive walrus analysis in this study could be due in part to the timing of feedings of the three different food types, as has been noted in captive sea lion studies (Deagle et al., 2005). It is uncertain why one scat sample from an individual captive walrus tested positive for decapods and sea cucumbers. Secondary prey consumption is one potential explanation for this anomalous finding (King et al., 2008) if fishes that were fed to captive walruses were harvested in locations where they may have fed upon these prey.

Ideally, strictly controlled experiments would be needed to assess likely causes for the observed variation and determine appropriate correction factors based on passage times and differential digestion of DNA (King et al., 2008). Given the broadly diverse diet of walruses in Alaska, many different prey types would be needed in this type of experimental study, making it logistically challenging and somewhat problematic with the number of combinations of dietary proportions necessary to replicate that seen among wild walruses. Nevertheless, amplification of prey DNA in the scats of pinnipeds and other marine mammals has been shown to be a valuable tool for assessing relative variations in their diets (e.g., Deagle et al., 2005; Ford et al., 2016; Schwarz et al., 2018).

Our analysis of diets of Bristol Bay walruses was based on a wide variety of known and potential prey that inhabit the region (Jewett & Feder, 1981; MacIntosh & Somerton, 1981; McDonald, Feder & Hoberg, 1981; Appendix B in Lauth, Dawson & Conner, 2019). Their diets consisted predominantly of benthic invertebrates as expected and were quite diverse as found in other regions (Sheffield & Grebmeier, 2009). This would suggest that walruses are adaptable to subsist on a wide variety of prey types throughout their range. While mollusks (bivalves and gastropods) are an important component of their diet in many locations, other invertebrate and fish species are also well represented in studies with large sample sizes (Sheffield and Grebemeier 2009), similar to our observations with more moderate sample numbers.

We did detect some variation in diets between our collection sites. Diet diversity was higher at Hagemeister Island, and especially Cape Seniavin, compared to Izembek and Round Island during both time periods. This may reflect spatial variation in diet, but could also reflect seasonal changes as collections at Hagemeister and Cape Seniavin were conducted in the autumn months, and in the summer months at the other sites. In Alaskan waters, diet diversity increased for Steller sea lions (*Eumetopias jubatus*) during autumn and winter compared to the breeding season (Fritz et al., 2019), as it did for female gray seals (*Halichoerus grypus*) in the northwest Atlantic Ocean (Beck et al., 2007). Lower diversity in summer may be related to a predatory focus on preferred prey that are subsequently reduced in abundance by autumn, leading walruses to diversify their diets as discussed further below.

The high occurrence and importance of polychaetes and tunicates found in Bristol Bay is different from the primary prey found in stomachs of male and female walruses from the northern Bering Sea, where bivalves and gastropods were more often seen in earlier decades (Fay & Stoker, 1982; Sheffield & Grebmeier, 2009). Our findings also contrast with a stomach content study of the four walruses collected near Cape Seniavin during April of 1981, which found that 90% of walrus prey biomass consisted of bivalves, predominantly *Spisula* and *Tellina* (Fay & Lowry, 1981). Although that sample size was small, those 1981 diet estimates were very different from our 2014–15 samples there and elsewhere, where *Spisula* ranked <5% in relative importance at all locations, and *Tellina* was not detected in our samples until 2017–18 at Round Island (Figs. 3 and 5). Additionally, polychaetes, gastropods, decapods, tunicates, and fishes appear to have had much greater presence in the diets of walruses in 2015 compared to 1981. These differences could be related to seasonal variations in diet (spring vs. autumn), an artifact of sample size (*n* = 4 vs. *n* = 17), differences in prey identification methodologies (stomach content vs. DNA analysis), variation among walruses, and location of foraging among trips. Yet, given that these differences are extensive, and that we observed little variation between our summer and autumn collections, major changes in the ecosystem between the 1980s and 2010s are also a credible explanation for these observations.

Another potential reason for these different findings between 1981 and the present study is a bias introduced by differential digestion of prey in walrus stomachs. That is, polychaetes are more rapidly digested than crustaceans or large clams and are therefore less likely to be identified in the earlier study based on stomach contents (Sheffield et al., 2001). Similarly, small clams may be digested more quickly than large clams (Sheffield et al., 2001) resulting in biases against small genera such as *Macoma* in stomach content studies. We found a substantial presence of *Macoma* clams in this study which were not a significant component of walrus diets in Bristol Bay in 1981 or elsewhere in the Bering Sea (Fay & Lowry, 1981; Fay & Stoker, 1982; Sheffield & Grebmeier, 2009)). The relative rate of digestion of tunicates, commonly found in the samples we analyzed, has not been studied to our knowledge. Tunicates have been a large part of the benthic biomass in the Bering Sea in some years (Lauth, Dawson & Conner, 2019).

However, we believe that differential rates of digestion of soft-bodied and harder-bodied prey is not a likely explanation for the absence of *Tellina* and very low abundance of *Spisula* in our samples in 2014–15. That is, had they been consumed, it seems likely that we would have detected their DNA. Therefore, although it is difficult to make direct comparisons between the present work and previous studies for variations in the diets of Pacific walruses, our findings suggest that changes may have occurred since the early 1980s.

It is also plausible that the differences we observed between diets at Cape Seniavin in September 2015 and from near there in April 1981 reported by Fay & Lowry (1981) were due to the effect of walruses on the prey base. Based upon the number of walruses hauling out on the north side of the Alaska Peninsula in the vicinity of Cape Seniavin, the number of days walrus spent in the area, their body mass, and their energy requirements, Fay & Lowry (1981) estimated that in 1980 they “could have consumed 17–33% of the total biomass of harvestable surf clams [*Spisula* ], or about two to four times the estimated annual sustained yield.” Thus, by September when our samples were collected, the walruses may have depleted *Spisula* and *Tellina* stocks to a degree that led them to broaden their diets to include greater proportions of other prey.

The PERMOVA results indicate the diet of walruses at Round Island in 2017–18 was significantly different than in 2015, although the small sample size in 2015 (*n* = 4) warrants some reservation to this conclusion. Nevertheless, the estimated diet at Round Island in 2015 was much more similar to other Bristol Bay locations from that year than it was to Round Island in 2017–18. Had more samples been collected at Round Island in the earlier years of this study, it is possible we would have identified some of these other species in the diets of walruses there as larger sample sizes tend to show a broader diversity of prey (Fay & Stoker, 1982; Sheffield & Grebmeier, 2009). Prey that were not detected in 2014–15 but appeared in 2017–18 included sipunculid burrowing worms (*Golfingia sp*.), *Tellina* and *Mya* clams, and the moon snail (*Euspira pallida*). These are all species that have been important components of the Pacific walrus diet elsewhere (Fay & Lowry, 1981; Fay & Stoker, 1982; Sheffield & Grebmeier, 2009).

Another interesting aspect of this study is the broad presence of hermit crabs (*Pagurus spp.*) in 2014–15, which were not found in the 2017–18 samples. Some *Pagurus* hermit crabs have a noted preference for the shells of moon snails (Jensen, 2014). Therefore, with the switch to moon snails in 2017–18, it is reasonable to assume that walruses were targeting the shells of this species and consuming what lay inside whether decapod or gastropod. Further investigations could reveal additional interesting predator–prey dynamics, such as the removal of moon snail bodies from their shells may provide habitat for hermit crabs in a sort of boom and bust scenario (Kellogg, 1976).

The significant difference in sample contents between Hagemeister Island and Cape Seniavin (both autumn collections) probably represents a degree of spatial variation in the benthos between northern and southern Bristol Bay. While the MDS showed a large amount of overlap in the walrus diets from those locations (Fig. 6), these differences appear to be related to greater importance of mollusks and lesser importance of fishes at Cape Seniavin compared to Hagemeister Island (Fig. 5). Yet, regardless of potential regional differences, a recent ecosystem-wide change does appear to have occurred based on the broader findings of this study and other work. The general similarity among collection sites in 2014–15 stands in stark contrast to collections made in 2017–18 (Fig. 6). A substantial reduction in the presence of *Lumbrineris* polychaetes, tunicates, and fishes in the recent years, coincident with increases in other species in walrus diets is indicative of this apparent shift. Additional changes are being seen in a broad spectrum of pelagic and benthic species throughout the Bering Sea and are associated with warming temperatures and low winter ice cover since at least 2013 Duffy-Anderson et al., 2019; Siddon & Zador, 2018; Stabeno et al., 2017; Stevenson & Lauth, 2019).

Significant ecosystem changes have been documented to occur within the southeastern Bering Sea with early seasonal ice retreat compared to years with late ice cover and are characterized by varying recruitment of copepod species and commercially important fishes (Hunt & Stabeno, 2002; Hunt et al., 2011). While commercially important species are well studied, but generally not detected in the diets of walruses in this study, little is known about variations in the abundance of non-commercially important benthic species that are preyed upon by walruses in this region during warm and cold periods. If the apparent temporal changes in walrus diets we found in this study were related to the change in environmental conditions from colder periods with later ice retreat to warmer periods with early ice retreat, they would suggest a two or three-year delayed response to a change in benthic community composition because the cold period ended by 2014 and our data indicate a change occurring between 2015 and 2017. Even so, changes in species assemblages associated with sea ice retreat can be non-linear and may result from a variety of cascading ecological factors and complex food-web dynamics that are not, as yet, fully understood in the Bering Sea (Mueter & Litzow, 2008). Continued monitoring of walrus diets in Bristol Bay will likely produce many insights into the effects of climate change and temporal lags on the community dynamics of benthic life in this region. Such monitoring would be especially useful if individual walrus diets were tied to tracked animals to make inferences about the distribution and abundance of prey.

## Conclusions

Walruses are a conspicuous and important element of the Bristol Bay ecosystem, which includes coastal residents who have a long cultural history in association with them. They also are intimately connected to the rich benthic community of this region and thus, by describing their diets, we have been able to identify a suite of species that play major roles in the contemporary ecology of Bristol Bay. We have further found evidence of changes in the relative abundances of several taxa of benthic fauna in the diet of Pacific walruses that coincide with, and might be related to, the pronounced warming of the Bering Sea in this century. The use of qPCR in this study provided a modern and effective means to detect and monitor change into the future. We acknowledge that additional captive and laboratory work is necessary to fine-tune diet estimation via appropriate correction factors. Even so, this study provides valuable contemporary information on vital aspects of walrus diets and this important ecosystem.

##  Supplemental Information

10.7717/peerj.8735/supp-1 Appendix S1 Forward and reverse DNA primers of potential walrus prey items designed for this study

10.7717/peerj.8735/supp-2 Supplemental Information 1 Inverse Ct scores of walrus diets from qPCR analysis

## Additional Information and Declarations

### Field Study Permissions

The following information was supplied relating to field study approvals (i.e., approving body and any reference numbers): Field collections were approved by the U.S. Fish and Wildlife Service (MA26132B-0).

### DNA Deposition

The following information was supplied regarding the deposition of DNA sequences: DNA sequences are available in the Supplementary Files and Appendix S1.

### Data Availability

The following information was supplied regarding data availability: Data and DNA sequences are available in the Supplementary Files. The sequences are also available at Genbank: Mallotus_villosus, MT122068; Chionoecetes_bairdi, MT122069; Clupea_harengus, MT122070; Hippoglossoides_elassodon, MT122071; Hippoglossus_stenolepsis, MT122072; Lepidopsetta_polyxystra, MT122073; Limanda_aspera, MT122074; Lycodes_palearis, MT122075; Pagurus_ochotensis, MT122076; Pleuronectes_quadrituberculatus, MT122077; Reinhardtius_hippoglossoides, MT122078; Tellina_lutea, MT122079.
