# Supplementary material for: Contemporary diets of walruses in Bristol Bay, Alaska suggest temporal variability in benthic community structure
Source: PeerJ. 2020 Mar 19;8:e8735. doi: 10.7717/peerj.8735 (PMC7085891; doi:10.7717/peerj.8735)
Supplement: Appendix S1 [file peerj-08-8735-s001.docx]

Appendix 1. Forward and reverse DNA primers of potential walrus prey items designed for this study.

| **Target** | **Forward primer 5' -3'** | **Reverse primer 5' - 3'** |
| --- | --- | --- |
| *Phoca* | CGACCTAGAAATTGCCCTACTC | CTTGCGGCTAGAAGAGAGATG |
| *Bivalvia* | GGACGAGAAGACCCT | CGCTGTTATCCCTATGGTAAC |
| *Gastropoda* | GGACCTGCCCAGTGAATATTTT | CAAACCATTCATACTAGCCTTCAATTAT |
| *Decapoda* | GACGATAAGACCCTA | CGCTGTTATCCCTAAA |
| *Pleuronictiformes & Perciformes* | AGACGAGAAGACCCT | CGCTGTTATCCCTAGGGTAAC |
| *Macoma calcarea* | GTAAGATTGAAAAGAAGAAGATAGCTACCA | GGTCGCAAACCTCCCATAAA |
| *Nuculana pernula* | ATTTACAGATTGGAGGTCGTTCCT | TCCATTCATTGGCCCACAA |
| *Spisula polynyma* | CCTATTGAGGGAAGCAAGCA | GCACCAAAGCGAGTCTTTAATC |
| *Tellina lutea* | GGCTGAAGGAAGGGACTAAA | GCTGTTATCCCTATGGTAACTATCT |
| *Serripes groenlandicus* | GCTCGTAGTTGGATCTCGATG | GTCAAGAGCACCAAGGGAATA |
| *Siliqua alta* | TTCGACCGCTGCGTACT | TAGACTCCTTGGTCCGTGTTT |
| *Hiatella arctica* | ATGGGCTAGTAAACGTCCTTATG | GATCCCTTTCACCACGGATTAG |
| *Mya truncata* | CCGACGTAGTTCTGACCATAAA | ACTTTGGTTTCCCGGAGTC |
| *Clinocardium ciliatum* | GGCTTTTTGTCCTCGTCCAA | TTCCGACAAAGCGACGGTAT |
| *Astarte spp.* | TTGGGCCTGCCAGTGAT | GGGCAAATTATTATGCTACCTTAGTACG |
| *Nucula tenuis* | GAGAAGACCCTGTTGAGCTTTA | TTACCTATTATCCCTCAGTTGCC |
| *Margarites spp.* | GACCTGCCCAGTGACTTATAGTTAAA | CCTCCAATAAAAAGGCAAATGATT |
| *Spisula spp. (clams fed at zoos)* | CAACGTGATACTGGCACAAAC | GTGACTTAGAGGTGTAGCTCTTG |
| *Neptunea ventricosa, heros & pribiloffensis* | TTAATCAAAAACATCGCTCCTTGTA | CGCGGCCGTTAAAAATATTC |
| *Neptunea lyrata* | GTTTCCCATCCTACGCTTCTC | TGCCTGCTTTGAACACTCTAA |
| *Buccinum spp.* | ACTTAGTACGAAAGGACCAGTTT | AGGAGTTTCACCTATATCTATTGATCC |
| *Euspira pallida* | GGACCTGCCCAGTGATTTAAAT | TTAAACCATTCATACTAGCCCTCAATT |
| *Pyrulofusus melonis* | GAGCACACCCCAAAACTTCTTT | CCGGATCACTTAGGTCAGAATTTC |
| *Pagurus trigonocheirus* | TAGAAACCAACCTGGCTCAC | GTCGCTATAAGAGAGAGTCTGTTC |
| *Pagurus ochotensis* | CGGCGTGGATATATAAATAAACTGT | GCTGTTATCCCTATGGTAACTTAATC |
| *Paralithodes camtschaticus* | CTGACTACCCTGATGCTTACAC | CAGATCGTGACGCCACTAAA |
| *Hyas coarctatus* | AAGAAGTTTGCGACCTCGAT | GGTCGAACAGACCCTCTTTAAT |
| *Chionoecetes opilio* | GATATACATAGTCTGGCCTGCTC | CCGACCATTCATACAAGCTTTC |
| *Erimacrus isenbeckii* | TGCTCACTGACATTTCTGTTTAAG | GTCCAATCATTCATACAAGTTCCC |
| *Pandalus spp.* | GAAAGTTCTTATCGACGGGAGTAG | CCACTCACTAAGGTGCTACAATTA |
| *Crangon spp.* | ACCGTGCTAAGGTAGCATAATC | AGGGTCTTGTCTTCCCATTAAA |
| *Limanda aspera  Hippoglossoides elassodon* | CTCCTTCCCCCGGTCAAT | CTCCATAGGGTCTTCTCGTCTTATG |
| *Microstomus pacificus* | CTGCCAAGGACCTGAACTAAA | GCTTGCGGGAGAAGAAGTAA |
| *Parophrys vetulus Lepisopsetta polyxystra* | CAGCTAAGGGCCTGAACTAAA | GCTTGCGGGAGAAGAAGTAA |
| *Acantholumpenus mackayi* | AAGTGGAACCTGCCCTAATG | GAGCTGTAGTTCTTAGTTGTAGGAG |
| *Ammodytes hexapterus* | AGAGGCCCAAGCTGATAGA | CTTACTCTCGGGTGCGTATAAC |
| *Anisarchus medius* | AAACATCGCCTCTTGCAAAATT | ATACCGCGGCCGTTAAACT |
| *Eumesogrammus praecisus* | CAGAAGCGGGAATACAAACATAAG | GGCAGGTTCCATTTGGTTTAAT |
| *Lumpenus fabricii* | CCGTTCTCCTTCTCCTATCTCT | GGGTCAAAGAAGGTGGTGTTA |
| *Hippoglossus stenolepsis Pleuronectes quadrituberculatus Reinhardtius hippoglossoides* | AACCCCCACGTGGAAAGG | TCTGCTGGTTAGAGCTGTTGCT |
| *Lycodes palearis* | ACCCCCACGTGGAATGG | CGGATCTTGTTGGTCAGAATTTC |
| *Clupea pallasii* | CGCCCACCAATCACGAA | CGAAGACGTTTGTGCCAGTATC |
| *Mallotus villosus* | GCCTGGCGTTCCGTTAATC | TCGCCCCAACCGAAGA |
| *Gammarus spp.* | ATTAGCTGTGCTCTCCGTAATC | GTCAAACCCGCACTCAAATG |
| *Styela rustica* | CTGCTGGGCATCTTCTTCTTA | TCGCTACCCTAATCTCCATAGT |
| *Echiurius echiurius* | GGCTGGCATGAATGGATTTAAC | GCGATAGGGTCTTCTTGTCTTC |
| *Nephtys spp.* | TCCTGCCCAGTGATTCATTAAA | ACTAGCCTCCAATTAAAAGGCAAA |
| *Lumbrineris spp.* | TTCCTGCCCAGTGATTCTATTTAAC | AAATCCGTTCATTCTAGCCTCTAATT |
| *Golfingia margaritacea* | CCTGCCCAGTGATTCTTTAACG | AAGGCGAAGGATTATGCTACCTT |
| *Priapulius caudatus* | GCTCATTTAGTCCCAGAAGGAA | AGTGTGATTGGGCGGATTAG |
| *Metridium senile* | TTAATTGTTGGCCGGTATGAATG | ACGTAGCATCTTCACTACGATTT |
| *Actiniaria* | GGGCAATCGCGGAGTATAAA | ACTTATTCCTTTGCTCGCTATGT |
| *Cucumaria calcigera* | GCCTGCCCAGTGGAAATTC | CCCATTTAAGAGACAAGTGATTATGC |
